# Community-acquired methicillin-resistant *Staphylococcus epidermidis* pyelonephritis in a child: a case report

**DOI:** 10.1186/1752-1947-8-415

**Published:** 2014-12-09

**Authors:** Hiroaki Kanai, Hiroki Sato, Yoshichika Takei

**Affiliations:** Department of Pediatrics, Suwa Central Hospital, Tamagawa 4300, Chino-city, Nagano, 391-8503 Japan

**Keywords:** Methicillin-resistant *Staphylococcus epidermidis*, Pyelonephritis, Vesicoureteral reflux, Antibiotic prophylaxis

## Abstract

**Introduction:**

*Staphylococcus epidermidis* is currently the most frequent pathogen of opportunistic and nosocomial infections worldwide. Most cases of *Staphylococcus epidermidis* infections are associated with indwelling medical devices and/or immunocompromised conditions. Community-acquired urinary tract infections are rare, particularly among pediatric populations, and clinicians often do not consider *Staphylococcus epidermidis* as a uropathogen.

**Case presentation:**

A previously healthy Japanese boy developed pyelonephritis caused by *Enterococcus faecalis* at 10 months of age. Subsequently, he was diagnosed with severe bilateral vesicoureteral reflux (right side grade V, left side grade III), and was administered trimethoprim/sulfamethoxazole as the prophylaxis. At 18 months of age, he presented with fever. Gram staining of urine obtained through catheterization revealed gram-positive cocci. We suspected pyelonephritis caused by enterococci, and administered oral fluoroquinolone empirically. The fever promptly resolved, and eventually, methicillin-resistant *Staphylococcus epidermidis* was detected at significant levels in the urine. Thus, our final diagnosis was pyelonephritis caused by community-acquired methicillin-resistant *Staphylococcus epidermidis*.

**Conclusions:**

Our case indicated that even immunocompetent children without a urinary catheter can develop *Staphylococcus epidermidis* pyelonephritis. *Staphylococcus epidermidis* can be underdiagnosed or misdiagnosed as sample contamination in community-acquired urinary tract infections. Therefore, when Gram staining of appropriately obtained urine samples reveals gram-positive cocci, clinicians should take into consideration not only the possibility of enterococci but also staphylococci, including *Staphylococcus epidermidis*, particularly in children with urinary abnormalities and/or those receiving continuous antibiotic prophylaxis.

## Introduction

*Staphylococcus epidermidis* (*S. epidermidis*) is one of the most common pathogens of nosocomial and opportunistic infections, and is generally associated with infections caused by indwelling foreign devices, including central vascular catheters, cerebrospinal fluid shunts or prosthetic cardiac valves, as well as by immunocompromised conditions [1, 2]. *S. epidermidis* is also the most common pathogen of nosocomial bacteremia in children, particularly in neonatal intensive care units, and is a common pathogen of healthcare-associated bacteremia in patients of all age groups [2]. Furthermore, prosthetic valve endocarditis, central venous catheter infections and cerebrospinal fluid shunt meningitis have also been documented to be caused by *S. epidermidis*[1]. However, community-acquired infections caused by *S. epidermidis* in immunocompetent children are rarely reported, and the etiology is unclear.

*S. epidermidis* was not reported as a uropathogen in recent studies of urinary tract infections (UTIs) [3–5]. Although information is limited and *S. epidermidis* infection may often be underdiagnosed or misdiagnosed as sample contamination, it appears evident that, overall, the prevalence of *S. epidermidis* in UTIs is extremely low. To the best of our knowledge, only six cases of pediatric UTIs caused by *S. epidermidis* have been reported [6–9].

Here, we report the case of an immunocompetent child receiving continuous antibiotic prophylaxis (CAP) with severe bilateral vesicoureteral reflux (VUR), who developed pyelonephritis caused by methicillin-resistant *S. epidermidis* despite having no urinary catheter.

## Case presentation

An 18-month-old Japanese boy with rapid onset high fever for the previous 12 hours presented to our emergency room. His perinatal and family history was unremarkable. There was no record of previous immunological problems.

**Figure 1:**
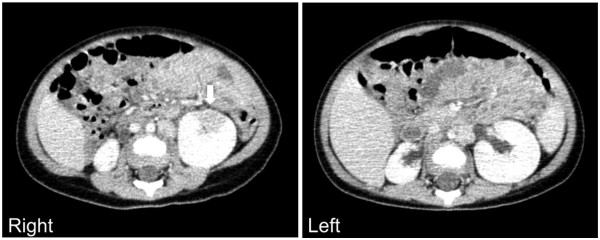
**Enhanced computed tomography findings indicating left pyelonephritis.** The right side image reveals decreased contrast enhancement in a part of the left renal parenchyma (arrow), indicating pyelonephritis. The left side image indicates the right small kidney and compensatory hypertrophic left kidney.

At 10 months of age, he was admitted to our hospital for examination owing to a fever of above 39°C, lasting for two days, for an unknown reason. Laboratory examinations revealed leukocytosis (white blood cell (WBC) counts: 20,410/μL), high absolute neutrophil counts (ANCs: 10,880/μL), and high levels of C-reactive protein (CRP: 6.39mg/dL). Although his urine analysis did not reveal pyuria, contrast-enhanced computed tomography revealed that a part of his left renal parenchyma showed decreased contrast enhancement, indicating pyelonephritis, which was also observed in the right small kidney and compensatory hypertrophic left kidney (Figure 1). He was thus diagnosed with pyelonephritis, and empirically treated with intravenous ceftriaxone (100mg/kg^-1^/day^-1^ at 24-hour intervals). Subsequently, a culture of urine obtained through catheterization revealed the presence of *Enterococcus faecalis*, which was susceptible to penicillins (Table 1). The urine was cultured, incubated on blood agar plates and bromothymol blue lactate agar plates at 37°C for species identification, and evaluated by slide culture for quantification of the organism. Moreover, we assessed the antimicrobial susceptibility of the isolate according to the Clinical and Laboratory Standards Institute criteria. The treatment was then changed to oral amoxicillin (60mg/kg^-1^/day^-1^ in three doses). He completed a three-week course of treatment and his symptoms were promptly resolved.

**Figure 2:**
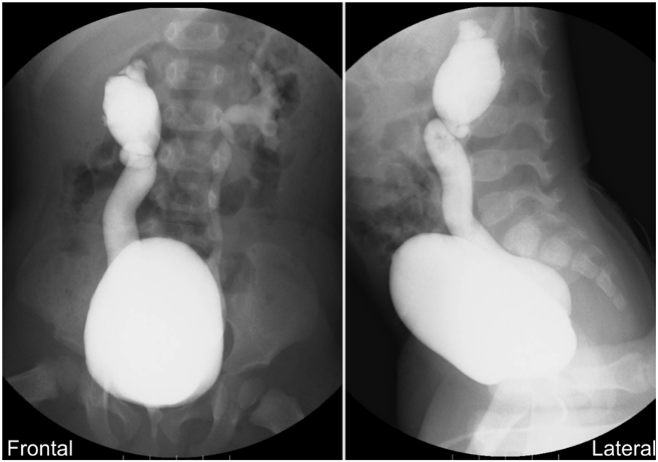
**Voiding cystourethrography findings.** Massive reflux of the right side, with significant ureteral dilatation and tortuosity and loss of the papillary impression, and reflux of the left side into a dilated ureter and blunting of the calyceal fornices were observed in both the frontal and lateral views. These findings led to the diagnosis of bilateral vesicoureteral reflux, right grade V, left grade III.

At 12 months of age, we performed a voiding cystourethrography, which showed severe bilateral VUR (right side grade V, left side grade III; Figure 2). Therefore, he was started on CAP with prophylactic-dose trimethoprim/sulfamethoxazole (TMP/SMX: 2mg/kg TMP and 10mg/kg SMX per day in one single dose); he did not develop recurrent UTIs for the next six months.

**Table 1:** **Antibiotic susceptibility for each uropathogen**

	First pyelonephritis	Second pyelonephritis
	***Enterococcus faecalis***	***Staphylococcus epidermidis***
Benzylpenicillin	S	R
Oxacillin		R
Ampicillin	S	
Sulbactam/ampicillin		R
Cefazolin		R
Cefotiam		R
Imipenem/cilastatin		R
Gentamicin		S
Amikacin	R	S
Levofloxacin	S	S
Minocycline	S	S
Vancomycin	S	S
Linezolid	S	S
Trimethoprim-sulfamethoxazole	R	S

S: susceptible, R: resistant.

When he presented to our hospital again at 18 months of age, his body temperature was 39°C. Other clinical examination findings were unremarkable. He had severe phimosis. The only abnormal laboratory findings were leukocytosis (WBC counts: 14,460/μL) and high ANCs (10,450/μL), but CRP levels were only slightly elevated (1.81mg/dL). The urine analysis revealed five to nine WBCs per high-power field and a remarkably elevated level of β2 microglobulin (1,490μg/L). Gram staining of urine obtained through catheterization revealed gram-positive cocci. While considering sample contamination, we also suspected pyelonephritis because there was no apparent source of the fever, and the urine sample was obtained by catheterization with a proper procedure. Moreover, during a routine examination two weeks prior, his urine analysis showed less than five WBCs per high-power field, despite the fact that it was bag-collected, and the β2 microglobulin level was within the normal range (195μg/L).

Since his clinical condition was generally good despite the high fever and he was not dehydrated, we determined that oral antibiotic treatment was possible. We planned to administer oral ampicillin after collecting blood and urine cultures, but we abandoned the treatment plan as he came to dislike the taste of oral amoxicillin subsequent to the prescription at first pyelonephritis at 10 months of age. Therefore, we selected tosufloxacin (oral fluoroquinolone, 12mg/kg^-1^/day^-1^ in two doses). The fever promptly declined one day after antibiotic therapy was started. After two days, we performed repeated blood examinations, which showed elevated CRP and WBC levels (12.2mg/dL and 16,140/μL, respectively), but significantly decreased ANCs levels (6,843/μL). In addition, we obtained a positive urine culture result indicating the presence of methicillin-resistant *S. epidermidis* (10^7^ colony-forming units per milliliter) as single bacterial species; the blood culture was negative for any bacterial organisms. Thus, our final diagnosis was pyelonephritis caused by community-acquired methicillin-resistant *S. epidermidis*, and, based on the result of the antibiotic susceptibility test, the antibiotics were adjusted to ‘therapeutic-dose’ TMP/SMX (10mg/kg TMP and 50mg/kg SMX per day in two doses; Table 1). The total antibiotic treatment duration was two weeks, and his fever did not reoccur. Because he had severe bilateral VUR and developed recurrent pyelonephritis despite receiving CAP, antireflux surgery was performed at 20 months of age. He has not had a reoccurrence of pyelonephritis to date.

## Discussion

*S. epidermidis* owes its pathogenic success to two major features: its natural niche on human skin, thus resulting in ready access to any device inserted or implanted across the skin, and its ability to adhere to biomaterials and form a biofilm [10, 11]. The organisms adhere to a prosthetic material and then form multilayered clusters that become embedded in an exopolysaccharide matrix, thus forming a biofilm. The biofilm protects the organisms from phagocytic cells and reduces the penetration of antibiotics, and thus appears to facilitate infection by shielding these normally low-virulent organisms from elimination by host defenses or antimicrobial therapy [3, 10, 11].

Treatment of *S. epidermidis* infections is generally difficult because of increasing resistance to many antibiotics. In particular, the number of strains showing resistance to methicillin, an antibiotic of first choice against staphylococci, has been increasing rapidly for many years [12]. Approximately 75 to 90% of hospital isolates of *S. epidermidis* show resistance to methicillin worldwide [13]. In addition to methicillin resistance, most *S. epidermidis* isolates are resistant to other antibiotics; most strains were found to be resistant to fluoroquinolones and macrolides, and many strains were resistant to clindamycin and TMP/SMX in North America and the United Kingdom [10]. Although this is mostly due to the high antibiotic resistance rates among nosocomial *S. epidermidis* isolates, treatment failure is also associated with the ability of *S. epidermidis* to form biofilms on inert surfaces of medical devices, increasing the difficulty of removal of these sticky, multilayered aggregates of bacteria [14].

In pediatric UTIs, *S. epidermidis* is rarely isolated; indeed, in a review of the English literature, we found only six reported cases (Table 2) [6–9]. In contrast to our case, all six reported cases first developed pyelonephritis caused by *S. epidermidis*, and no precedent antibiotics had been administered. None of the cases, including our case, involved a urinary catheter, and all were immunocompetent. In addition, all but one of the patients were preadolescents. It is noteworthy that all cases had urinary abnormalities; four were severe VUR (Grade III and above), and although the other two did not have reflux, they did have had bladder diverticulum, which can lead to urine stasis. These previous reports and the details of our case demonstrate that a UTI caused by *S. epidermidis* can occur in individuals of all ages, even in immunocompetent children, and all susceptible patients have apparent underlying urinary tract abnormalities. Although no detailed description of the susceptibility pattern was provided and methicillin-resistant *S. epidermidis* was not specified in previous reports, antibiotics other than penicillins and cephalosporins are considered necessary in such cases.

**Table 2:** **Literature review of pediatric cases of urinary tract infections caused by** ***Staphylococcus epidermidis***

Source	Age (years)	uWBCs (/hpf)	Sensitive	Resistance	Urinary abnormalities
Hagler et al. 1990 [6]	9	>100	Cephalosporins, VCM, TMP, EM, nitrofurantoin	ABPC	No VUR, Bladder diverticulum
	11	20-30	EM, nitrofurantoin, tetracycline	ABPC	No VUR, Bladder diverticulum
Hall et al.1994 [7]	6	None	VCM, TMP/SMX, clindamycin	ABPC, cephalosporins	VUR: Bilateral Gr III, Duplications
	7	3-5	Cephalosporins	-	VUR: Right Gr III, Left Gr II Posterior urethral valve
McDonald et al. 1994 [8]	11	5-10	-	-	VUR: Right Gr III
Upadhyayula et al. 2012 [9]	0.6	5-10	VCM, TMP/SMX, GM	Ceftriaxone	VUR: Right Gr V, Left Gr IV
Present	1.5	5-9	VCM, TMP/SMX, GM, amikacin, minocycline, levofloxacin	Penicillins, cephalosporins	VUR: Right Gr V, Left Gr III Right small kidney

ABPC: ampicillin; EM: erythromycin; GM: gentamycin; hpf: high power field; Gr: grade; TMP/SMX: trimethoprim/sulfamethoxazole; uWBCs: urine white blood cells; VCM: vancomycin; VUR: vesicoureteral reflux.

The pathogenesis of *S. epidermidis* UTIs in previously reported cases and our case remains unclear. Numerous studies have clearly indicated that the ability to form biofilms on inert surfaces represents a typical feature of nosocomial infections. Bacteria adhere to a surface by unspecific factors, such as hydrophobicity and surface charge, and the initial adherence stage is followed by accumulation of the biofilm [11, 12]. As mentioned above, our case did not have a urinary catheter at presentation and no evidence of an immunological problem. Therefore, based on previous reports, severe VUR associated with dilated ureters may somehow be a predisposing risk factor for *S. epidermidis* infection. Moreover, it is noteworthy that in our case, *S. epidermidis* was susceptible to TMP/SMX, even though our patient had previously received CAP with prophylactic-dose TMP/SMX. Our patient had already experienced pyelonephritis caused by enterococci, which was resistant to TMP/SMX; however, we selected TMP/SMX as the antimicrobial prophylaxis to prevent UTIs caused not by enterococci, but by gram-negative rods, which are primary uropathogens even in recurrent UTIs or severe VUR. According to these conditions, we speculated that the presence of bilateral dilated ureters causing persistent urinary stasis allowed for *S. epidermidis* to produce a protective biofilm and enhanced its adhesion to the mucosal surface of the ureters. In addition, the most likely explanation for why our patient developed pyelonephritis caused by *S. epidermidis* susceptible to TMP/SMX regardless of receiving CAP with TMP/SMX is as follows. Normally, the simple and compound papillae in the kidney have an antireflux mechanism that prevents urine in the renal pelvis from entering the collecting tubules. However, some VURs result in intrarenal reflux, and subsequently, the infected urine stimulates an immunologic and inflammatory response in the kidney parenchyma, causing pyelonephritis [15]. In addition, as *S. epidermidis* is surrounded by a biofilm, it can resist phagocytosis, and further impair the penetration of many antibiotics. Consequently, although the organisms were susceptible to TMP/SMX *in vitro*, the prophylactic effect might become weak *in vivo*.

In reviewing the clinical course of our case report retrospectively, we have highlighted the main factors contributing to the potential for underdiagnoses or misdiagnoses of *S. epidermidis* pyelonephritis. Although our patient did not show any symptoms other than high fever and urine analysis indicated pyuria by definition, the nitrate test results were negative and the CRP level as the serum inflammatory marker was only slightly elevated. Therefore, we would not have suspected pyelonephritis at initial presentation from these examination results alone. However, Gram staining of urine samples obtained aseptically through catheterization showed significantly positive gram-positive cocci, which indicated the possibility of pyelonephritis. Thus, we empirically decided to administer antibiotics. Two days later, his CRP levels were markedly elevated to 12.2mg/dL, despite the fact that his fever was resolved, and ANCs decreased. Ultimately, a single *S. epidermidis* identified from the urine specimen obtained aseptically through catheterization was found to grow at a rate of 10^7^ colony-forming units per milliliter. On the basis of these findings and the antimicrobial treatment response, we confirmed that *S. epidermidis* was the uropathogen. Fortunately, we could select the antibiotics appropriate for methicillin-resistant *S. epidermidis*. If we had initially considered that the presence of *S. epidermidis* was due to sample contamination, appropriate therapy, including suitable antibiotics for a sufficient duration and the subsequent surgery, would not have been provided. Furthermore, inappropriate or insufficient antibiotic treatment might have caused urosepsis or renal damage. Our case indicates that in ambulatory practice, opportunistic pathogens like *S. epidermidis* have the potential to be underdiagnosed or misdiagnosed, thereby increasing the risk of treatment failure.

With regard to laboratory findings of pyelonephritis, leukocytosis, neutrophilia and elevated CRP level are common in the acute phase of pyelonephritis [15]. However, these are nonspecific markers of bacterial infection, and their elevated levels do not prove acute pyelonephritis [15]. Although our patient first revealed leukocytosis and neutrophilia at the second pyelonephritis, his CRP level was not significantly elevated (1.81mg/dl). However, two days after presentation, his CRP level was remarkably elevated (12.2mg/dl), while the clinical symptoms improved. Some clinical studies have examined the significance of laboratory data, including CRP, to diagnose acute pyelonephritis in children (Table 3) [16–19]. These studies indicated that CRP levels have a wide range and are not always elevated, even in acute pyelonephritis. In addition, it was shown that although the specificity of CRP was low, the sensitivity was relativity high. Also, these studies had important limitations. Despite it being well known that CRP level is affected by the timing of examination, the time of blood examinations from the onset was not described. Thus, as seen in our patient, low CRP levels (especially lower than 2mg/dl) could not exclude acute pyelonephritis, and it was considered that CRP was only one of the ’predictive’ but poor ‘diagnostic’ markers of pyelonephritis.

**Table 3:** **The levels of C-reactive protein (CRP) at the time of diagnosis, sensitivity, specificity, positive (PPV) and negative predictive values (NPV) of CRP for diagnosis of acute pyelonephritis in children (cut-off values of 2mg/dl)**

Reference number	N	Mean ± SD, mg/dl	Sensitivity, %	Specificity, %	PPV, %	NPV, %
Smolkin et al. 2002 [16]	42	12	100	18.5	30.9	100
Pecile et al. 2004 [17]	53	10.6 ±2.6	94.4	31.9	61.4	83.3
Nikfar et al. 2010 [18]	62	ND	80	65	79	67
Xu et al. 2014 [19]	21	6.82 ±3.94	85.71	48	50	80

ND: not described.

Yet, it remains a difficult challenge to differentiate true *S. epidermidis* infection from contaminants. This is because *S. epidermidis* occupy a prominent position in the commensal flora of the human skin and mucous membranes and are thus frequently encountered as culture contaminants. Regarding bacteremia, approximately 1 to 6% of blood cultures are contaminated, and coagulase-negative staphylococci (usually *S. epidermidis*) are responsible for between 70 and 80% of such cases [10]. However, in UTIs, the epidemiology of contaminants is not apparent. Therefore, it is important for clinicians to be aware of the possibility of ‘true’ *S. epidermidis* infection when it is identified in urine culture, even in immunocompetent cases or in cases without indwelling medical devices, and not simply presume that the bacteria represent contamination. A variety of clinical and laboratory parameters, including urine collection techniques, images of urinary tracts, and the subsequent response of antibiotic treatment should be examined and evaluated in such cases. Based on the clinical course of our case and the literature review, we suggest that urinary tract abnormalities are a risk factor for *S. epidermidis* infection in pediatric UTIs. Further studies are needed to identify the prevalence of *S. epidermidis* UTIs in children and the factors responsible for developing the UTI.

## Conclusions

Our case raises three important considerations. First, even immunocompetent children without a urinary catheter can develop pyelonephritis caused by methicillin-resistant *S. epidermidis*. Second, because *S. epidermidis* is one of the most commonly diagnosed contaminants in various clinical settings, it can easily be underdiagnosed or misdiagnosed in pediatric UTIs if an accurate urine culture is not obtained using catheterization or suprapubic aspiration. Third, clinicians should consider not only enterococci but also staphylococci as potential uropathogens when Gram staining of appropriately obtained urine reveals the presence of gram-positive cocci, particularly in children with urinary abnormalities and/or CAP.

## Consent

Written informed consent was obtained from the patient’s legal guardian for publication of this case report and accompanying images. A copy of the written consent is available for review by the Editor-in-Chief of this journal.

## Abbreviations

*S. epidermidis*: *Staphylococcus epidermidis*
UTIs: urinary tract infections
CAP: continuous antibiotic prophylaxis
VUR: vesicoureteral reflux
WBC: white blood cell
ANCs: absolute neutrophil counts
CRP: C-reactive protein
TMP/SMX: trimethoprim/sulfamethoxazole.
